# Stipa pennata subsp. ceynowae (Poaceae, Pooideae), a new taxon from Central Europe

**DOI:** 10.3897/phytokeys.83.12797

**Published:** 2017-07-17

**Authors:** Ewelina Klichowska, Marcin Nobis

**Affiliations:** 1 Institute of Botany, Jagiellonian University, Kopernika 27, PL-31-501 Krakow, Poland

**Keywords:** feather grasses, micromorphology, numerical analyses, Poland, taxonomy

## Abstract

Based on numerical analyses of macromorphological characters, scanning electron microscopy observation of leaves and lemma micromorphology, as well as field observations, Stipa pennata subsp. ceynowae was described here as a new taxon from Poland. It differs from the most similar S. pennata subsp. pennata and *S. borysthenica* mainly by its longer ligules of vegetative shoots. The affinities of this taxon are discussed and a morphological comparison with related species is provided. Illustrations and images of the micromorphological structures, as well as information about its distribution, habitat and conservation status are given.

## Introduction

*Stipa* Linnaeus (1753) is one of the largest genera in the family Poaceae, subfamily Pooideae (Soreng et al. 2015). In the narrow approach, it comprises over 150 species distributed in open grasslands and steppes, with the highest species diversity in the warm temperate regions of the Old World (Roshevitz 1934, Tzvelev 1968, 1976, Bor 1970, Martinovský 1980, Freitag 1985, Wu and Phillips 2006, Nobis 2013). New species of *Stipa* continue to be described. For instance, in the last twenty years, over thirty species have been described from such countries as Morocco, Spain, Italy, Turkey, Kazakhstan, Kyrgyzstan, Tajikistan, Mongolia, Bhutan, India and China (e.g. Kotukhov 1998a, 1998b, Noltie 1999, Vázquez and Ramos 2007, Vázquez et al. 2009, Nobis 2013, 2014a, Vázquez and Gutiérrez 2011, Zhao and Guo 2011, Cataldo et al. 2012, Tzvelev 2012, 2014, Nobis et al. 2013, 2016a). At the same time, there are also still many unresolved taxonomic problems within the different sections and taxonomic complexes of this genus.

One of the numerous and taxonomically problematic sections in the genus *Stipa* is the nominal section, which comprises (depending on the approach) from 15 to 55 species (Smirnov 1925, Martinovský 1965, 1970, 1977, 1980, Klokov and Osychnyuk 1976, Tzvelev 1976, 1986, Freitag 1985, Vázquez and Gutiérrez 2011, Gonzalo et al. 2013, Nobis et al. 2016b). In Central Europe (including Czech Republic, Germany, Poland, Slovakia, Hungary and Austria), the section Stipa is represented by about 10 taxa: *S. bavarica* Martinovský and Scholz (1968), *S. borysthenica* Klokov ex Prokudin (1951), *S. dasyphylla* (Ćernjaev ex Lindemann 1882) Trautvetter (1884), *S. eriocaulis* Borbás (1878) subsp. eriocaulis, S. eriocaulis subsp. austriaca (Beck von Mannagetta 1890) Martinovský (1965), *S. pennata* Linnaeus (1753), *S. pulcherrima* Koch (1848), *S. styriaca* Martinovský (1970), *S. tirsa* Steven (1857) and *S. zalesskii* Wilensky ex Smirnov (1925) (conf. Martinovský 1980, Tzvelev 1986, Conert 1998, Marhold and Hindák 1998, Danihelka et al. 2012). In Poland, there are only four species from the above-mentioned section, namely: *S. borysthenica*, *S. pennata*, *S. pulcherrima* and *S. eriocaulis*, all of them reaching here the northwestern limit of their general range (Ceynowa-Giełdon 1976, Ceynowa-Giełdon et al. 2014a, 2014b, Nobis 2014b, Nobis et al. 2017). The section Stipa can be divided into many critical groups of closely related and morphologically similar taxa. One example is the group that includes *Stipa pennata*, a species which over the years has undergone numerous changes. Before Freitag (1985) chose a lectotype from the original material studied by Linnaeus, the name *S. pennata* was regularly used by various authors to identify different species. The most correct seems to be Mansfeld’s (1939) assumption of the synonymization of *S. joannis* Čelakovský (1884) with *S. pennata*. Furthermore, some authors distinguished within the group a number of species and many units of lower rank (Klokov and Osychnyuk 1976, Tzvelev 1976, 2006, Martinovský 1977, 1980), whereas others distinguished a single species and several taxa of lower rank (Freitag 1985).

In our work within the *S. pennata* group, we included taxa previously classified in the series *Penicilliferae* Martinovský (1976) and characterized by having leaves with apical tassel, ventral line of hairs terminating below the top of lemma, dorsal line free and longer than the subdorsal ones. Within series *Penicilliferae*, Martinovský (1977) recognized four species: *S. joannis* (= *S. pennata*), *S. borysthenica*, *S. styriaca* Martinovský (1970) and *S. danubialis* Dihoru and Roman (1969). *Stipa styriaca* and *S. danubialis* are endemic species (Martinovský 1977) respectively for Austria and Romania. There are five additional taxa from Asia that fit the criteria for incorporation to ser. Penicilliferae (sect. Stipa): *S. kirghisorum* Smirnov (1925), *S. turkestanica* Hackel (1906) subsp. turkestanica, S. turkestanica subsp. trichoides (Smirnov 1925) Tzvelev (1974), *S. macroglossa* Smirnov (1924) subsp. macroglossa and S. macroglossa subsp. kazachstanica (Kotukhov 1994) Nobis (2013). Taxonomic revision as well as macro- and micromorphological variation of those aforementioned taxa have recently been presented by Nobis et al. (2016b).

During the taxonomic revision of the central European representatives of the *Stipa pennata* group, we came across herbarium specimens from Folusz near Szubin in Poland that greatly differ from the hitherto known species. On the basis of these sheets, Ceynowa-Giełdon (1976) distinguished Stipa joannis var. cujavica. Unfortunately, the name of this taxon was not validly published because the author provided only its brief description in Polish with no references to the type and place of its preservation. The aim of our study was to examine distinctiveness of individuals from Folusz in relation to other Central European taxa from *S. pennata* group by using multivariate morphometric analysis.

## Materials and methods

Over 500 herbarium sheets with specimens from the *Stipa pennata* group deposited at B, FRU, GAT, GOET, JE, KFTA, KHOR, KRA, KRAM, LE, LECB, M, MSB, MW, NY, PE, POZ, SZUB, PR, PRC, TAD, TASH, TK, TRN, UPS, W, WA, WU were examined (acronyms by Thiers 2016). The morphological characteristics of the vegetative and generative structures were examined on well-developed specimens. For numerical analysis, we selected 177 herbarium sheets (67 of *Stipa borysthenica*, 104 of *Stipa pennata*, and 6 of *Stipa* from Folusz). A list of the morphological characteristics used in analyses is presented in Table 1. Measurements were taken using a Nikon SMZ800 stereo microscope.

In accordance with the assumption of numerical taxonomy (Sokal and Sneath 1963), each specimen was treated as an Operational Taxonomic Unit (OTU). For testing the normal distribution of each characteristic, the Lilliefors and Shapiro–Wilk statistical tests were performed. Those that did not fulfill the criterion of normality were log-transformed. Next, the Pearson correlation coefficient was calculated; the characteristics in which a strong correlation was found (>0.9) were excluded from further analyses. To illustrate the relationship between the studied taxa and also to select the features that best describe the existing variability, a Principal Component Analysis (PCA) was conducted using all quantitative characteristics. According to the Kaiser criterion, factors with eigenvalues >1 were chosen (Kaiser 1960) and characteristics with the highest factor loadings of the first three principal components (r≥0.60) were determined. Subsequently, descriptive statistics of characters for all recognized taxa were calculated. Levene’s test was using to assess the equality of variances. To reveal significant differences between means of particular characters across all examined taxa, one-way analysis of variance (ANOVA) and nonparametric Kruskal-Wallis test followed by Tukey’s HSD test or multiple comparison test were calculated. All statistical analyses and calculations were performed using Statistica software, version 10 (Statsoft Inc. 2011).

For observations in a scanning electron microscope, samples were coated with gold using a JFC-1100E Ion sputter manufactured by JEOL, then observed and photographed using a Hitachi S-4700 scanning electron microscope (SEM). The methods and terminology were adopted from Thomasson (1978, 1981), Ellis (1979), Snow (1996) and Nobis (2013, 2014a).

**Table 1.** Morphological characters used in the present analyses, involving *Stipa pennata* group.

Abbreviation	Character
AL	length of anthecium (mm)
AwnL	length of awn (mm)
CL	length of callus (mm)
Col1L	length of lower segment of awn (mm)
Col2L	length of middle segment of awn (mm)
CRL	length of peripheral ring of callus base (mm)
CRW	width of peripheral ring of callus base (mm)
DDL	distance from the end of dorsal line of hairs to the top of anthecium (mm)
DVL	distance from the end of ventral line of hairs to the top of anthecium (mm)
LigC	length of ligules of the middle cauline leaves (mm)
LigIV	length of ligules of the internal vegetative shoots (mm)
LC	length of culm (mm)
LCL	length of upper cauline leaves (mm)
LP	length of panicle
LV	length of vegetative shoots (mm)
NF	number of flowers in panicle
SL/ColL	ratio length of seta to the sum of length of lower and middle segment of the awn
WA	width of anthecium (mm)

## Results

### Numerical analysis

**Figure 1.**
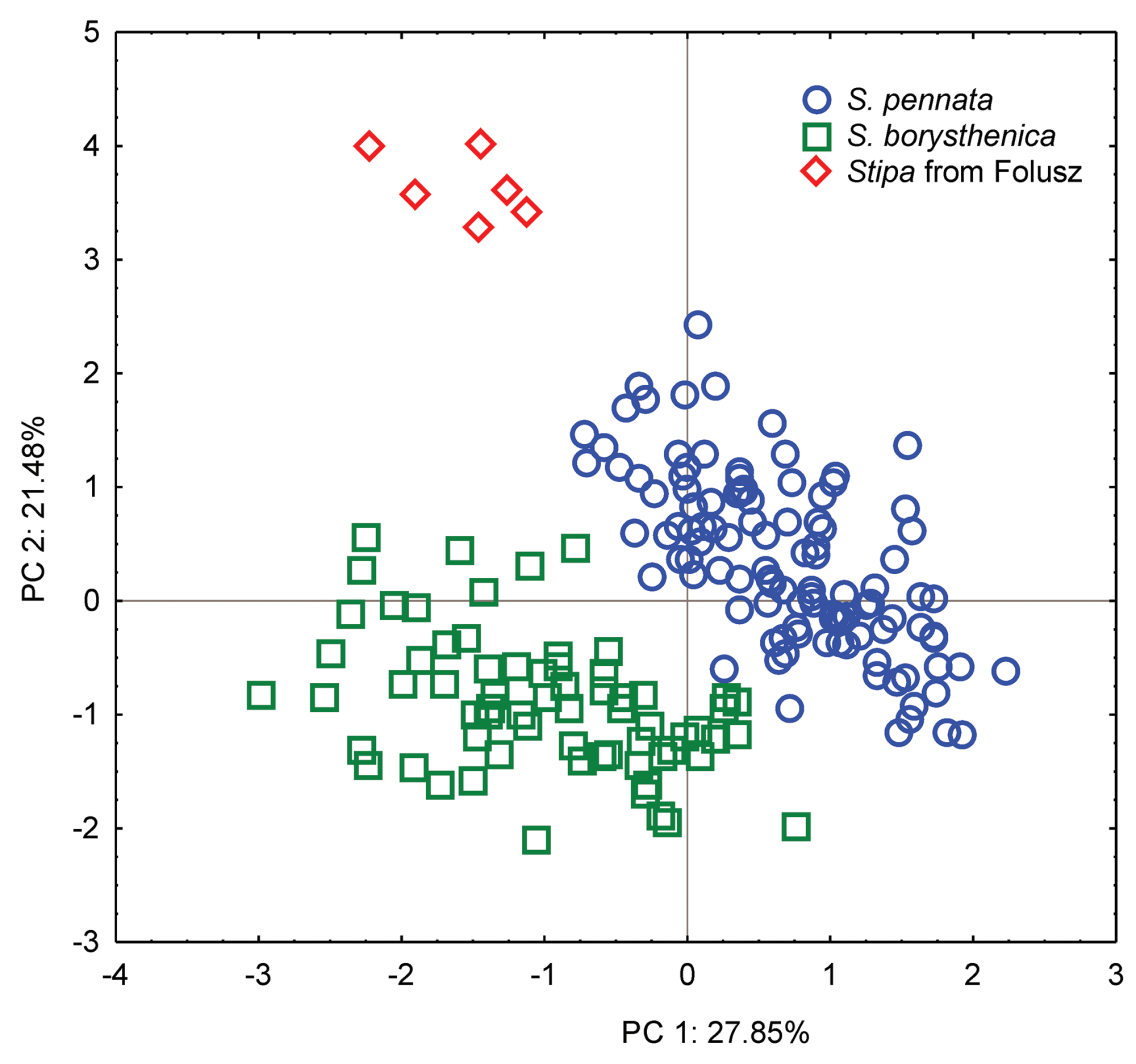
Biplot of principal component analysis (PCA) performed on 18 characters.

The result of the Principal Component Analysis (PCA) revealed twelve characteristics with high factor loadings (r≥0.6) on the first three principal components. Together, the first three components accounted for 57.71% of the total variation. The first two components explained respectively 27.85% and 21.48% of the total variation (Table 2). The scatterplot of the first two axes showed three group of points (Figure 1). Seven characteristics, including AL, CL, CRL, LCL, LigC, LP, and NF, displayed the highest correlations with the first axis, grouping specimens of *Stipa borysthenica* on the left and *S. pennata* on the right side. The remaining characteristics (AwnL, Col_1_L, LC, LigIV, LV) highly influenced the second axis, separating specimens representing *Stipa* from Folusz.

The results of the one-way ANOVA/Kruskal-Wallis test revealed significant differences in all examined characters (Table 2). The results of the post hoc tests (Tukey’s HSD test for variables with normal distribution and multiple comparison tests for characters with non-normal distribution) are presented in Table 3. The ranges of variability of the most important characteristics of the designated morphological groups corresponding to the two examined taxa and the population from Folusz are presented in Table 4.

**Table 2.** Results of numerical analysis involving *Stipa pennata* group. Principal component analysis (PCA): factor loadings for 18 characters, eigenvalues and percent variation. The highest factor loadings (≥ 0.6) are bolded. One-way ANOVA: F and p values for characters with normal distribution. Kruskal-Wallis test: H and p values for characters with non-normal distribution. The highest F/H values are bolded. For characters abbreviations see Table 1.

Character abbreviation	PC 1	PC 2	PC 3	ANOVA	
				F/H	p
AL	-**0.77**	0.24	0.33	20.28	0.000
AwnL	-0.41	**0.68**	-0.04	19.01	0.000
CL	-**0.81**	-0.34	0.27	**248.95**	0.000
Col_1_L	0.23	**0.72**	0.41	**47.26**	0.000
Col_2_L	-0.24	0.58	0.45	15.27	0.001
CRL	-**0.62**	-0.07	0.21	**53.73**	0.000
CRW	0.55	0.37	-0.16	**64.72**	0.000
DDL	-0.50	0.48	0.13	15.29	0.001
DVL	-0.21	0.19	0.38	7.20	0.027
LC	-0.38	**0.65**	-0.39	**64.24**	0.000
LCL	-**0.62**	-0.36	-0.01	17.41	0.000
LigC	-**0.66**	-0.14	-0.13	**113.10**	0.000
LigIV	-0.37	**0.60**	-0.16	**118.02**	0.000
LP	-**0.76**	-0.33	-0.11	**100.61**	0.000
LV	-0.23	**0.74**	-0.34	28.80	0.000
NF	-**0.67**	-0.35	-0.03	**79.41**	0.000
SL/ColL	-0.57	-0.16	-0.57	11.76	0.001
WA	-0.11	0.48	-0.29	21.46	0.000
Eigenvalue	5.01	3.87	1.51		
Percent variation (%)	27.85	21.48	8.38		

**Figure 2.**
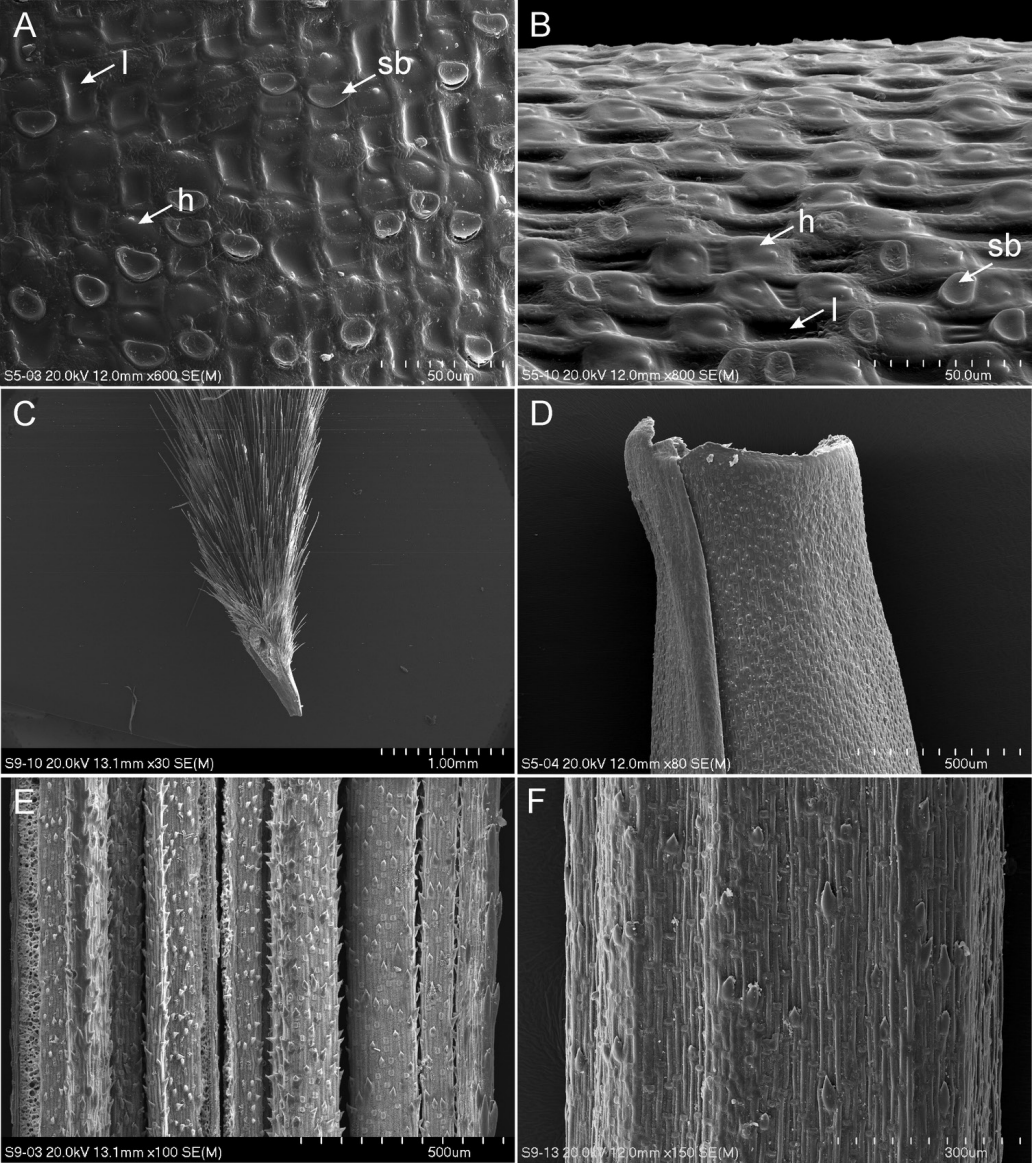
SEM morphology of *Stipa* from Folusz. **A** Structure of lemma – superior view **B** Structure of lemma – lateral view **C** Callus **D** Top of anthecium **E** Adaxial surface of vegetative leaves **F** Abaxial surface of vegetative leaves. Abbreviations: h = hook, l = long cell, sb = silica body.

**Table 3.** Results of post-hoc tests. Tukey’s HSD test for characters with normal distribution, multiple comparison tests for characters with non-normal distribution. + – statistically significant, p < 0.05; ns – not significant. *Stipa pennata* – pe, *Stipa borysthenica* – bo, *Stipa* from Folusz – F. For characters abbreviations see Table 1.

Character	pe-bo	pe-F	bo-F
AL	+	+	ns
AwnL	ns	+	+
CL	+	+	+
Col_1_L	+	+	+
Col_2_L	ns	+	+
CRL	+	+	ns
CRW	+	ns	+
DDL	ns	+	+
DVL	ns	ns	ns
LigC	+	+	ns
LigIV	ns	+	+
LC	ns	+	+
LCL	+	+	ns
LP	+	ns	ns
LV	+	+	+
NF	+	ns	ns
SL/ColL	+	ns	ns
WA	+	+	+
Number of significance differences	12	13	10

**Table 4.** Main morphological differences among selected members of *Stipa pennata* group. Measurements are given in millimeters.

Taxon Character	*S. borysthenica*	S. pennata subsp. ceynowae	S. pennata subsp. pennata
Anthecium length	(15.7–)17.00–18.9(–20.4)	(17.4–)18.1–19.9(–20.0)	(14.25–)15.9–18.0(–19.8)
Awn length	(225–)279–334(–396)	(305–)328–412(–442)	(228–)283–340(–408)
Callus length	(3.4–)3.7–4.2(–4.6)	(3.1–)3.3–3.8	(2.4–)2.8–3.25(–3.75)
Column length	(43–)57–69(–59)	81–91(–94)	(55–)64–78(–93)
Ligules of the middle cauline leaves length	(1.2–)2.2–4.2(–6.3)	(2.6–)2.8–4.3(–4.4)	(0.4–)1–2.5(–4.0)
Ligules of internal vegetative shoots length	(0.9–)1.3–2.2(–3.4)	(3.2–)4.1–5.2(–6.7)	(1.0–)1.3–2.2(–3.6)
Uppermost cauline leaves length	(22–)36–62(–125)	(16–)27–38(–69)	(4–)10–22(–40)
Shape of callus base	Cuneate	Piriformis	Piriformis

### Scanning microscope observation

The results of SEM observations showed that the general patterns of the lemma micromorphology of *Stipa* from Folusz are typical for the genus *Stipa* (cf. Barkworth and Everett 1987, Nobis 2013, Nobis et al. 2013, 2016b) (Figure 2). Fundamental (long) cells are elongated, rectangular to a more or less square in shape. The side walls of long cells are raised and undulate, but often hidden under a thick layer of wax that hinders observation. Silica bodies are quite common, reniform to oblong or ovate, while cork cells are sparse or absent. Hooks are frequent, oriented towards the lemma apex, whereas prickles are completely absent. Lemma apex is glabrous (Figure 2A–D).

The adaxial surface of leaves of the vegetative shoots is ribbed and densely covered by short prickles, long cells and silica bodies (Figure 2E). Whereas the abaxial surface is dominated by long cells with admixtures of silica bodies and sparsely distributed prickles (Figure 2F).

### Taxonomic treatment

Conducted analysis clearly indicated that specimens from Folusz represents a new taxon, which is described below.

#### Stipa pennata subsp. ceynowae

Taxon classificationPlantaePoalesPoaceae

Klichowska & M.Nobis subsp. nov.

urn:lsid:ipni.org:names: 77164173-1

**Figure 3.**
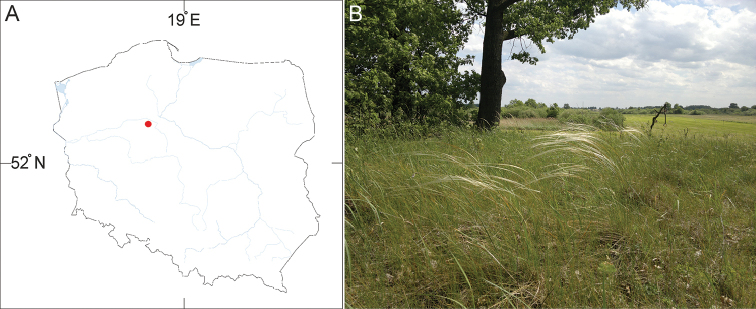
Stipa pennata subsp. ceynowae from Folusz near Szubin (Poland). **A** Map of distribution in Poland, red dot – locality of population **B** Photograph of habitat.

**Figure 4.**
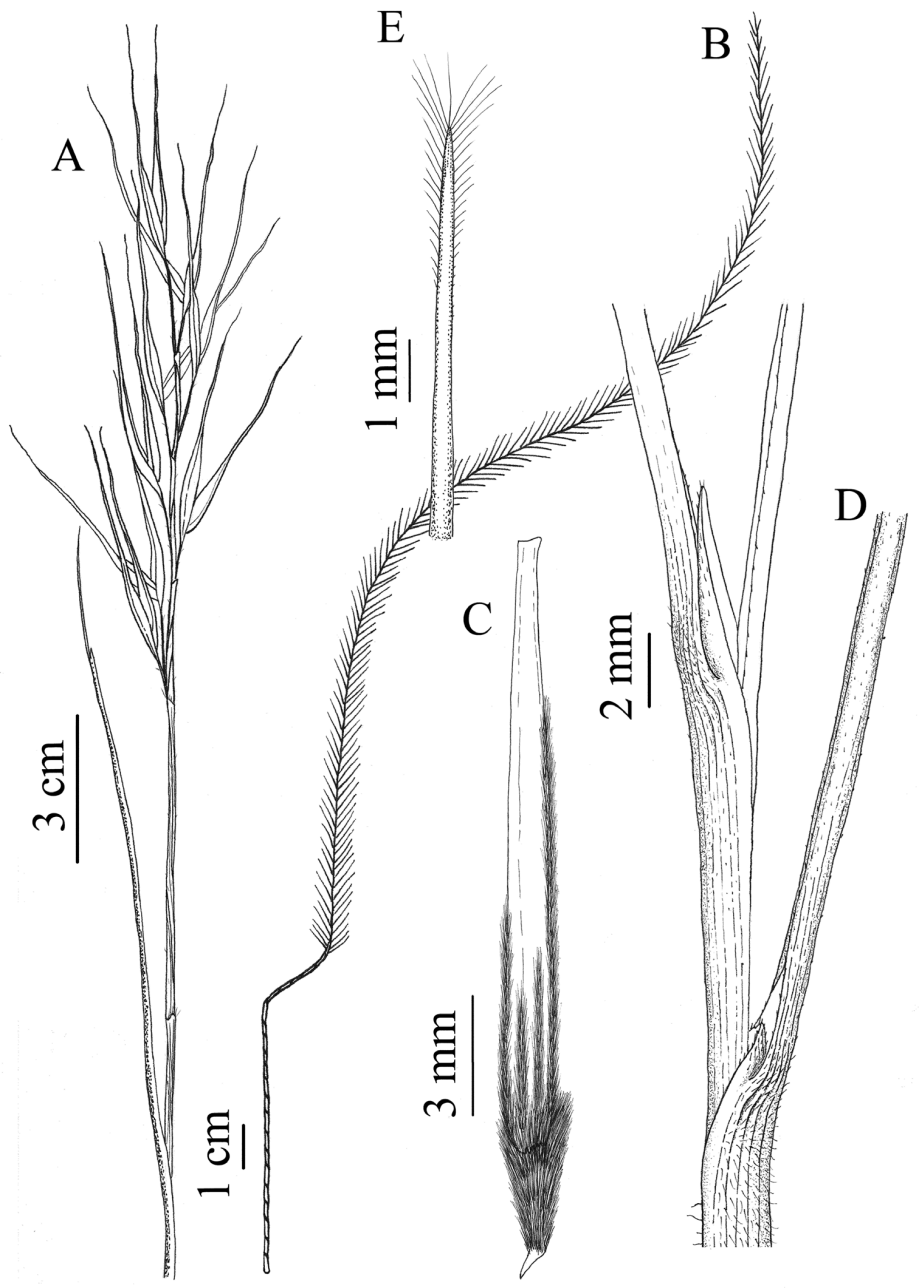
Stipa pennata subsp. ceynowae based on the holotype. **A** Panicle with upper cauline leaves **B** Awn with glabrous column and pilose seta **C** Anthecium **D** External (the lower) and internal (the upper) ligules of the vegetative leaves **E** Apex of juvenile leaves with an apical tassel of hairs.

**Figure 5.**
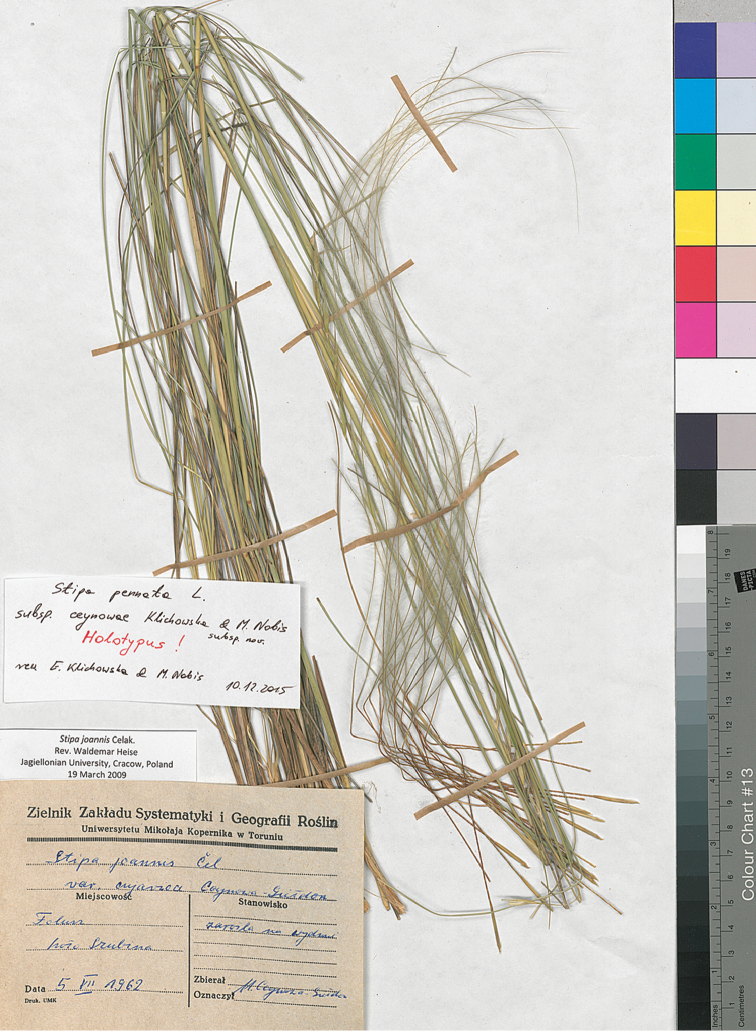
Holotype of Stipa pennata subsp. ceynowae.

Figures 2 , 3 , 4 , 5

##### Diagnosis.

Stipa pennata subsp. ceynowae is most similar to S. pennata subsp. pennata from which differs mainly in longer ligules of internal leaves of vegetative shoots (3.2–)4.1–5.2(–6.7) mm vs. (1.0–)1.3–2.2(–3.6) mm and lemmas with a somewhat longer awn (305–)328–412(–442) mm vs. (228–)283–340(–408) mm respectively.

##### Type.

POLAND. Folusz koło Szubina, zarośla na wydmie [Folusz near Szubin, scrub on the dune], 5 July 1962, *Ceynowa-Giełdon s.n.* (holotype TRN!, isotype KRA 0451189!).

##### Description.

Plant perennial, densely tufted, with a few culms and numerous vegetative shoots. Culms (56–)84–95(–99) cm tall, 3–4-noded, glabrous at nodes and slightly scabrous to more or less densely pubescent below them. Leaves of vegetative shoots: sheaths of external leaves shortly pilose to scabrous, of internal leaves scabrous to almost glabrous; ligules membranous, acute or slightly obtuse, with very short cilia at the apex and shortly setulose on the back, of external leaves (1.0–)1.1–2.1(–2.7) mm long, of internal leaves (3.2–)4.1–5.2(–6.7) mm long; blades convolute, green to pale green, (73–)81–97(–107) cm long, (0.5–)0.7–0.8(–1.0) mm in diameter, abaxial surface from scabrous, covered by short spinules (on external leaves) to slightly scabrous or almost smooth, with spinules almost confined to the margins of leaf blades (on internal leaves), adaxial surface always covered by short prickles less than 0.1 mm long, juvenile leaves usually with an apical tassel of hairs up to 2 mm long. Cauline leaves: sheaths smooth to slightly scabrous (usually in upper part of sheath); ligules of the middle cauline leaves (2.6–)2.8–4.3(–4.4) mm long, slightly acute or obtuse, at the apex with very short cilia, and with short bristles on the back; blades convolute, green or pale green, the uppermost one (1.6–)2.7–3.8(–6.9) cm long, abaxial surface scabrous. Panicle 10–11(–14.5) cm long, contracted, with 8–10 spikelets; branches scabrous or with short hairs 0.2–0.5(–0.8) mm long. Glumes subequal, 56–64 mm long, narrowly lanceolate. Anthecium (17.4–)18.1–20.0 mm long and 1.0–1.25 mm wide; callus (3.1–)3.3–3.8 mm long, with hairs (1.6–)1.7–2.1(–2.4) mm long in ventral part and (1.0–)1.1–1.4 mm long in dorsal; foot of callus curved, peripheral ring flattened 0.9–0.95 × 0.3–0.35 mm; lemma straw-coloured, with 7 lines of hairs, dorsal and subdorsal lines slightly fused at the base, ventral line with (0.5–)0.6–0.8 mm long hairs, terminating at 1/2–2/3 of lemma length about 4.5–5.8(–6.4) mm below the top of lemma; dorsal line with (0.4–)0.5–0.6 mm long hairs, terminating at 1/3 of lemma length, about (9.1–)9.5–11.0(–11.1) mm below the top; awn (305–)328–412(–442) mm long, bigeniculate; column smooth and glabrous, twisted, straw-coloured or slightly green, 0.5–0.6 mm wide near base, 81–91(–94) mm long with the lower segment of column (63–)65–69(–70) mm long and the upper (19–)22–24 mm long; seta (222–)247–318(–354) mm long, pilose, with 5.2–6.0 mm long hairs, gradually decreasing in length towards apex; palea straw-coloured equaling lemma in length. Caryopsis ca.12 mm long.

##### Etymology.

The name of taxon honors the collector—Prof. Mirosława Ceynowa-Giełdon, who first noted the distinctiveness of *Stipa* individuals from Folusz.

##### Distribution and habitat.

Stipa pennata subsp. ceynowae is an endemic taxon, known only from Folusz settlement near Szubin in Kuyavia region (northern Poland). It grows on a dune hill surrounded by wet meadows occurring in the Gąsawka River Valley. The subspecies occurs on small fragment of dry, sandy grassland adjoining oak and pine stands. At the locality, the following species grow together with *Stipa*: *Achillea pannonica* Scheele, *Asperula tinctoria* L., *Avenula pratensis* (L.) Dumort., *Betula pendula* Roth, *Calamagrostis epigejos* (L.) Roth, *Carex praecox* Schreb., *Dianthus carthusianorum* L., *Euphorbia cyparissias* L., *Festuca trachyphylla* (Hack.) Krajina, *Filipendula vulgaris* Moench, *Galium verum* L., *Geranium sanguineum* L., *Peucedanum oreoselinum* (L.) Moench, *Poa pratensis* L., *Polygonatum odoratum* (Mill.) Druce, *Vincetoxicum hirundinaria* Medik.

##### Phenology.

Flowering period: May–June.

##### Conservation status.

*Stipa pennata* is a species protected in Poland (Regulation of the Minister of the environment dated October 9, 2014) as well as it was included in the Polish red data book of plants (Ceynowa-Giełdon et al. 2014b). The only known locality of S. pennata subsp. ceynowae was partly destroyed by the extraction of sand (up to the mid-1950s) and the subsequent afforestation of pine and birch trees carried out in the 1990s (Ceynowa-Giełdon 2001, Nienartowicz et al. 2014). Currently, S. pennata subsp. ceynowae should be considered as a critically endangered (CR) species—to date, only several flowering individuals have survived (tufts with 8, 10, 11 and 14 culms), occupying a very small area of dry grassland. Lack of grazing has resulted in increased ground cover by layer of “steppe felt”, which hamper seeds germination and seedlings growth. Also, tree seedlings pose a threat by shading the grasslands. Similarly, as in the case of other dry grassland species— survival depends on the preservation of suitable habitat conditions, which can be achieved through active protection. Due to the extremely small size of the population, it seems reasonable to apply the methods of *ex situ* conservation, including *in vitro* propagation.

**Additional specimens studied (paratypes).** POLAND. Folusz, 16 Jun 1959, *Michalska and Bohr s.n.* (TRN!); Folusz koło Szubina nad Gąsawką, na wydmie, wśród łąk [Folusz near Szubin on the Gąsawka River, on a dune, among meadows], 13 Jun 1972, *Ceynowa-Giełdon s.n.* (TRN!)×4; North Poland, Kuyavian-Pomeranian Voivodeship, Folusz near Szubin by the Gąsawka River; xerothermic grassland on a sandy dune, 3 Jun 2014, *Klichowska s.n.* (KRA 0451190!).

## Discussion

Ceynowa-Giełdon (1976) distinguished Stipa joannis var. cujavica (*nom. inval.*) based on the longer hairless part of the awn, longer vegetative leaves and longer upper cauline leaves than in the case of the typical variety. Although the Principal Component Analysis supports the usefulness of these characteristics (Table 2), their larger size can also be found in individuals of S. pennata subsp. pennata and, after examining a great number of individuals, they seem to be insufficient to distinguish this taxon based on its description. According to our results, the internal vegetative leaves (Figure 4D) in specimens of S. pennata subsp. ceynowae have distinctly longer ligules (usually 4.1–5.2 mm in length) than the other closely related species from Poland, namely *S. borysthenica* and S. pennata subsp. pennata (in both cases, usually reaching of 1.3–2.2 mm in length; Table 4). Our research carried out on a large number of herbarium specimens (from the geographical range of these taxa), as well as on the findings of other authors (Bor 1970, Tzvelev 1976, Martinovský 1977, 1980, Conert 1998, Gonzalo et al. 2013, Nobis et al. 2016b), confirm that all known taxa closely related with *S. pennata* do not have such long ligules of their vegetative shoots. Ligules of a similar length or even longer are observed in other species of the section Stipa occurring in Central Asia, namely: S. turkestanica subsp. turkestanica, S. macroglossa subsp. macroglossa and S. macroglossa subsp. kazachstanica (Gonzalo et al. 2013, Nobis et al. 2016b). However, S. pennata subsp. ceynowae cannot be confused with any of them due to its definitely longer anthecium, callus, awn, culm and vegetative leaves, as well as to its distribution, limited only to Central Europe.

*Stipa kirghisorum*, is another species that is morphologically similar to both S. pennata subsp. pennata and S. pennata subsp. ceynowae. However, *S. kirghisorum* differs from the two above-mentioned taxa by the strongly scabrous abaxial surface of leaves of the vegetative shoots, shorter anthecium (13.1–)14.5–16.0(–17.8) mm and ventral line of hairs terminating (0.5–)1.4–3.1(–4.6) mm below the top of the lemma, as well as its general range that is limited to the Central Asia (Nobis et al. 2016b).

Stipa pennata subsp. ceynowae is somewhat similar to two other European species from ser. Penicillifera. First is *S. styriaca* that is also characterized by having long awn up to 445 mm and anthecium 17.5–21.5 mm, but in contrast to S. pennata subsp. ceynowae it has densely pubescent leaf sheaths (with 0.2–0.8 mm hairs) (Martinovský 1977). The second species is *S. danubialis* that differs from Stipa pennata subsp. ceynowae by having pilose column (lower part of awn) and anthecium 23–25 mm long (Martinovský 1977).

Due to its long awn, Stipa pennata subsp. ceynowae could be also confused with *S. pulcherrima* that occurs in Central Europe too. However, it can easily be distinguished by its ventral lines of hairs terminating at 1/2–2/3 of lemma length, shorter anthecium (17.4–20.0 mm) and longer ligules on vegetative shoots, while *S. pulcherrima* is characterized by ventral lines reaching the base of the awn, anthecium 18–25 mm long and ligules of the vegetative shoots not exceeding 2 mm long (Martinovský 1980, Nobis 2014b).

The results of ANOVA and post-hoc tests confirm separateness of the taxon from Folusz (Tables 2, 3). Stipa pennata subsp. ceynowae differs from S. pennata subsp. pennata and from *S. borysthenica* in a statistically significant way by 13 and 10 characters respectively (Table 3).

### A key to identification of feather grasses (*Stipa*) in Poland

**Table:** 

1	Awns scabrous throughout	***S. capillata***
–	Awn smooth in the lower pat and plumose in the upper	**2**
2	Ventral line of hairs on lemma not reaching the base of awn, ending (1.0–)3.0–6.0(–7.9) mm below the top; dorsal line only in lower 1/4 of its length fused with subdorsal ones	**3**
–	Ventral line of hairs on lemma reaching the base of awn; dorsal line at least in 3/4 of its length fused with subdorsal ones	**5**
3	Blade of uppermost cauline leaf (22–)36–62(–125) mm long; floret callus (3.4–)3.7–4.2(–4.6) mm, straight to slightly curved, callus base cuneate	***S. borysthenica***
–	Blade of uppermost cauline leaf (4–)10–24(–69) mm long; floret callus (2.4–)2.8–3.3(–3.8) mm long, curved, callus base piriform	**4**
4	Ligules of internal leaves of vegetative shoots (1.0–)1.3–2.2(–3.6) mm long; column of awn (55–)64–78(–93) mm long; blade of uppermost cauline leaves (4–)10–22(–40) mm long	***S. pennata*** subsp. ***pennata***
–	Ligules of internal leaves of vegetative shoots (3.2–)4.1–5.2(–6.7) mm long, column of awn 81–91(–94) mm long; blade of uppermost cauline leaves (16–)27–38(–69) mm long	***S. pennata*** subsp. ***ceynowae***
5	Leaves of the vegetative shoots distinctly scabrous; anthecium (18.1–)20.6–22.8(–24.6) mm long; floret callus (3.7–)4.4–5.1(–5.8) mm long; awn (277–)328–394(–463) mm long	***S. pulcherrima***
–	Leaves of the vegetative shoots glabrous and smooth to very slightly scabrous especially in their lower part; anthecium (15.0–)16.3–18.7(–20.7) mm long; floret callus (3.4–)3.6–4.4(–5.0) mm long; awn (218–)228–269(–312) mm long	***S. eriocaulis***

## Supplementary Material

XML Treatment for Stipa pennata subsp. ceynowae
